# A letter

**DOI:** 10.4103/0970-1591.57907

**Published:** 2009

**Authors:** P. Venugopal

**Affiliations:** 801, Symphony Apartment, Sturrock Road, Mangalore - 575 001, India

Dear Editor,

It is nice to get up in the morning with good news after what has been happening over the past 1 month. The entire Urology community was dumped into a gloomy atmosphere. I am sure this happy news will prompt all of us to come out of the lethargy and the desire to publish will kindle in most of us.

Indian Journal of Urology (IJU) was started in 1984 with Mahendra Bhandari as its first editor when I was the President of Urological Society of India (USI). The genesis for the need for a journal started in 1976 at the Bangalore Conference when the then Governing Council pronounced the idea as premature. It took another 8 long years for us to be able to proudly boast of a journal and the first issue was released at the Kolkata Conference in 1984. Though we had considerable difficulty in the initial years, due to the tenacity of many, IJU weathered the storm. Credit has to be given to the subsequent Editorial team for nudging IJU forward.

Nitin and his team have to be complimented for the untiring efforts to bring IJU forward to enable it to be considered for acceptance in PubMed. All articles from 2008 are now available with Pubmedcentral as well. This is indeed a great stride that we have achieved.

It has taken 25 years to achieve this. Time is as important as gradually enhancing the quality of IJU. I have a habit of browsing many journals, Urological or otherwise. In one of my write-ups earlier, I had mentioned that I found IJU much better than many of the so called high-rated journals especially with regard to Urology. If one were to peruse some of the common Urological Journals one will find many articles that carry no weight being published. Either they are company sponsored without mentioning conflict of interest or may be due to some other reason. IJU is definitely head and shoulders above such journals.

We have achieved one laurel but should not rest there. Continuous progress should be maintained. Now that we have achieved this, I am sure many of the Urologists in this country and other countries will turn to IJU to have their articles published. We already have many from abroad publishing in IJU (as Nitin said many are invited). We have many in this country who refrain from publishing their work in IJU. When enquired some responded that IJU is not indexed and they prefer their articles in Indexed Journals. Now that IJU is in PubMed, this excuse cannot be used anymore. If we as Indian Urologists want our journal to prosper, then we have to send all our material to IJU for publication. One of the reasons for publishing in other journals is said to be the time taken for seeing it in print. This can be rectified if the volume of articles sent in for publication increases, then maybe IJU will consider additional issues than what we have now. I am sure all Urologists from India will start sending their materials to be published in IJU and consider other journals only in the unlikely event of not gaining entry into IJU.

The next goal for IJU is to climb the ladder with regard to impact factor. With many excellent articles published in IJU, I am sure the day is not far for us to attain higher rating with impact factor. All it needs is our cooperation and active support.

I wish we could persuade Nitin and his team to continue for at least another term. At this stage we can not afford to lose continuity. I am sure Nitin and his team will hear our plea and accept to continue. They have taken IJU to this height and I along with several others feel it is there bounden duty to take it to the top. I do agree that no one can remain in a post for eternity. Changes are needed to infuse fresh thoughts. It is time to groom the next Nitin. It will take time. One retrograde step at this juncture will undo all that we achieved so far.

I would like to give my sincere thanks to Nitin and his team for taking us this far and pray and hope that we will reach the pinnacle of fame in the near future.

With warmest regards to all,

